# Does previous open or percutaneous renal stone surgery affect retrograde intrarenal surgery outcomes?

**DOI:** 10.3906/sag-2007-76

**Published:** 2021-06-28

**Authors:** Özer GÜZEL, Can AYKANAT, Yılmaz ASLAN, Ahmet ASFUROĞLU, Melih BALCI, Altuğ TUNCEL

**Affiliations:** 1 Department of Urology, Ankara Numune Research and Training Hospital, University of Health Sciences, Ankara Turkey

**Keywords:** Kidney stone, retrograde intrarenal surgery, percutaneous nephrolithotomy, open renal surgery

## Abstract

**Background/aim:**

Open or percutaneous renal stone surgery can have an adverse effect on the collecting system of the kidney. We evaluated retrograde intrarenal surgery outcomes in patients with ≤30 mm renal stones who had open or percutaneous renal stone surgery history.

**Materials and methods:**

A total of 707 patients who underwent retrograde intrarenal surgery treatment were included in this study. Fifty-six patients had open or percutaneous renal stone surgery history (Group 1) and the remaining did not (Group 2, n = 651). The groups were compared in terms of age, stone size, stone-free rates, and complications.

**Results:**

The mean age of the patients was 51.16 ± 14.8 and 45.95 ± 14.6 years in Groups 1 and 2, respectively (p = 0.008). The mean stone size was 14.97 ± 6.1 mm and 16.47 ± 6.9 mm in Groups 1 and 2, respectively (p = 0.107). The stone-free rates were 71.4% and 84.1% in Group 1 and 2 respectively and it was significantly higher in Group 2 (p = 0.013). The overall rate of postoperative complications was higher in Group 1 (p = 0.019), but there was no difference between the two groups in terms of Clavien 1–2 and 3–4a complication rates.

**Conclusion:**

Our results showed that having a history of open or percutaneous renal stone surgery has a negative effect on the success and complication rates in retrograde intrarenal surgery. Therefore, patients should be well informed before this operation.

##  1. Introduction. 

The high global prevalence of urolithiasis (3%–5%), recurrence of the disease, and factors playing a role in its etiology, such as lifestyle, physical inactivity, and unfavorable dietary habits have placed a greater burden on the economy and health services of countries worldwide [1]. A study by De et al. from the United States of America reported that the dietary changes alone have led to an increase in the prevalence of urolithiasis from 3.8%–8.8% [2]. In Turkey, the prevalence of this condition was reported to be 11.1% [3]. Several factors, such as genetics, sex, age, occupation, geography, dietary habits, climate, and seasonal changes are known to play a role in the etiology of urolithiasis [4]. 

The treatment methods for this health problem that is relevant to a significant part of the society aim to achieve a complete stone-free state with the lowest morbidity rate. While the treatment was limited to open surgery during the pre‐Shock Wave Lithotripsy (SWL) period, minimally invasive methods have been developed since the introduction of SWL in the 1980s, and this led to the wide adoption of percutaneous nephrolithotomy (PCNL) and ureterorenoscopy (URS), as well as an increased use of mini‐ and micro‐PCNL with retrograde intrarenal surgery (RIRS) [5]. RIRS has been proposed as an alternative to PCNL or SWL in the treatment of renal stones due to its high stone free rates and low complications [6–8]. 

Although open or percutaneous renal surgery was the main surgical treatment method before RIRS, the lower rate of complications and higher percentages of success achieved by RIRS gradually reduced the use of these surgical procedures [9]. 

 Furthermore, open or percutaneous renal stone surgery can have an adverse effect on the collecting system of the kidney. In the literature, there are only few reports on the use of RIRS for the treatment of renal stones in patients with a history of PCNL or open surgery. In this study, we evaluated the safety and efficacy of single session RIRS in patients with ≤30 mm renal stones who have open or percutaneous renal stone surgery history.

The introduction should argue the case for the study, outlining only the essential background, and should not include the findings or the conclusions. It should not be a review of the subject area, but should finish with a clear statement of the question being addressed.

## 2. Material and methods

A total of 758 patients with ≤30 mm renal stones that underwent RIRS treatment at our clinic between September 2013 and January 2018 were retrospectively reviewed. The study was performed in accordance with the most recent version of the Declaration of Helsinki and informed consent was not required because of the retrospective nature of the study. Fifty-one patients with renal anomalies, such as ectopic kidney, hypotrophic kidney uretero-pelvic junction obstruction, calyceal diverticula, or a duplicated urinary system and patients who had undergone RIRS as second look immediately after PCNL or open stone surgery were excluded from the study. We included the patients who had visualized the collector system on spiral CT and had no abnormal structural defects. As a result, the study was conducted with a total of 707 patients. Of the 707 patients, 56 had a history of open or percutaneous renal stone surgery (Group 1), and the remaining patients did not have a history of renal stone surgery (Group 2, n = 651). 

The demographic and clinical characteristics of the patients, age, sex, affected side, localization, stone size, stone-free rates, and complications were noted. Preoperative tests, such as complete blood count (CBC), coagulation studies, serum biochemistry, urine culture, and plain X-ray were conducted. Spiral computerized tomography (CT) scans were obtained routinely to assess the characteristics and location of the stones. Stone size was determined by measuring the longest axis on preoperative imaging modalities. In cases of multiple renal calculi, stone size was defined as the sum of the greatest dimensions of each stone. Stone attenuation measured as Hounsfield unit (HU) on noncontrast CT. Preoperative antibiotics were administered for prophylaxis. Patients who had a positive urine culture were given appropriate antibiotics according to the antibiogram results.

Continuous general anesthesia was used in all the patients. The procedures were performed in the lithotomy position. RIRS was performed using 7.5-Fr flexible ureteroscope (Karl Storz Flex-X2, Karl Storz, Tuttlingen, Germany) through a 9.5-Fr ureteral access sheath (Cook, Cook Medical, Dublin, Ireland). The stones were fragmented (1.5 J x 8 Hz) or dusted (0.5 J x 20 Hz ) with a 30W Holmium:YAG laser device (SphinxX, Lisa, Katlenburg-Lindau, Germany) until the fragments were small enough to pass out spontaneously. During the procedure, irrigation solution was applied to the desired location via gravity.

Stone-free status was evaluated at 46 weeks after the single session operation with low-dose contrast enhanced spiral CT. Operative success was defined as a complete stone-free status or residual stone of ≤3 mm on imaging methods. The two groups were compared with regard to age, stone size and location, stone-free rate, operative parameters, postoperative outcomes, and complications. Postoperative complications were recorded according to the modified Clavien–Dindo Classification [10] based on the following four grades: Grade 1 includes minor risk events not requiring therapy (with exceptions of analgesic, antipyretic, antiemetic, and antidiarrheal drugs or drugs required for lower urinary tract infection). Grade 2 refers to potentially life-threatening complications with the need of intervention or a hospital stay longer than twice the median hospitalization for the same procedure. Grade 3 complications are defined as those requiring surgical, endoscopic or radiological intervention. Life-threatening complication (including CNS complications)‡ requiring IC/ICU-management defined as Grade 4 complications and is further divided into two subgroups based on the invasiveness of the therapy selected to treat the complication; Grade 4a including single organ dysfunction (including dialysis) and Grade 4b including multi organ dysfunction. Grade 5 indicates death of a patient due to a complication. 

### 2.1. Statistical analysis 

Statistical analysis was performed using Statistical Package for the Social Sciences (SPSS) v:16.0 for Windows (SPSS Inc., Chicago, IL, USA). The comparison of continuous variables between two groups were done with either Mann–Whitney U test or student t test according to distribution normality test. The comparison of categorical variables were done with Fisher exact or Chi-square test where applicable. Odds ratio calculated for the history of open or percutaneous renal stone surgery by using the Chi-square test. P-value __? was considered statistically significant.

## 3. Results

The overall mean age of the 707 patients was 46.36 ± 14.7 (14–91) years, being 51.16 ± 14.8 (21–75) years in Group 1 and 45.95 ± 14.6 (14–91) years in Group 2 (p = 0.008). The sex distribution was 443 males (62.7%) and 264 females (37.3%). In Group 1, the mean time after the first operation was 7 ± 3.6 years (1–18 years). The stones were located in the renal pelvis in 417 patients (59.0%), upper pole calices in 34 (4.8%), middle pole calices in 126 (17.8%), and lower pole calices in 130 (18.4%). Of the stones 23 (41.1%) in group 1 and 122 (19%) in group 2 were multiple. There was no staghorn stone in both groups. The mean Hounsfield unit (HU) of the stones was 1034 ± 645 and 985 ± 745 HU in Groups 1 and 2, respectively (p = 0.226).

The mean stone size was 15.09 ± 6.1 (4–30) mm and did not significantly differ between Groups 1 and 2. Access sheath was used in 90.6% of Group 1 and 92.1% of Group 2 (p = 0.580). The mean operation time were 60.8 ± 12.5 and 55.4 ± 14.4 min, in Group 1 and 2 respectively (p = 0.604). A JJ stent was placed in 96.9% patients at the end of the operation based on the surgeon’s preference. The stent was removed four weeks after the surgery. The operative results are summarized in Table 1. The mean stone-free rate were 83.3% in all patients. The SFR rates was lower in Group 1 than the Group 2 (71.4% vs. 84.1%, p = 0.013). Residual stones were found in 16 of the 56 patients (28.6%) in Group 1 and 102 of the 651 patients (15.7%) in Group 2, which indicated a significantly higher rate in Group 1. The odds ratio (OR) was 2.01 (95% confidence interval [CI], 1.10–3.63, p = 0.02) for the history of open or percutaneous renal stone surgery. The stone size was classified as 0–10 mm, 10–20 mm, and 20–30 mm, and the stone-free rate was found to decrease as the stone size increased in Group 2. Nevertheless, group 1 patients had lower stone free rate in all diameter of stone size when compared to Group 2. However, the stone-free rate didn’t show statistically significant difference within Group1 and Group 2 patients according to stone-size. According to the stone size (0–10mm, 10–20mm, 20–30mm), the SFR rates in Group 1 and 2 were 80.0%, 75.0%, 53.8% (p = 0.284) and 96.2%, 82.6%, 62.0% (p = 0.446) respectively.

**Table 1:** Patient characteristics.

Parameters	Group 1	Group 2	Overall	p
Number of patients	56	651	707	
Median age ± SD (range) year	51.16 ± 14.8 (21–75)	45.95 ± 14.6 (14–91)	46.36 ± 14.7 (14–91)	0.008*
Sex, n· Male · Female	39 1	404 247	443 (62.7%)264 (37.3%)	0.260
Affected side· Right· Left· Bilateral	22 32 2	296 338 17	318 (45.0%)370 (52.3%)19 (2.7%)	0.643
Localization · Pelvis· Upper calyx· Middle calyx· Lower calyx	29 2 8 17	388 32 118 113	417 (59.0%)34 (4.8%)126 (17.8%)130 (18.4%)	0.117
Stone size ± SD (range) mm	14.97 ± 6.1(4–30)	16.47 ± 6.9 (5–30)	15.09 ± 6.1 (4–30)	0.107
Stone size distribution (n)· 0–10 mm· 10–20 mm· 20–30 mm	15 28 13	234 317 100	249 (35.3%)345 (48.8%)113 (15.9%)	0.198

* Mann–Whitney U test

The mean duration of hospitalization was 1.1 ± 1.3 (0–20) and 1.4 ± 1.8 (0–12) days in Groups 1 and 2, respectively (p = 0.2).

Postoperative complications were observed in 2.0% of the patients (n =14). In Group 1, three patients had grade 1–2 complications (JJ stent migration in one and fever in two), and one patient had a grade 3–4a complication (septicemia) requiring intensive care. In Group 2, 7 patients had grade 1–2 complications (perirenal hematoma in one, stent migration in one, and fever in five), and three patients had grade 3–4a complications (septicemia in two and prolonged fever in one requiring intensive care). According to our results, the overall postoperative complication rate was higher in Group 1 (p = 0.019), but there was no difference between the two groups in terms of Clavien 1–2 and 3–4a complication rates. Clavien > 4a complication was not seen in any of the patients. Complications of the groups are summarized in Table 2.

**Table 2:** Operative outcomes of the two groups.

Parameters	Group 1 (n:56)	Group 2 (n:651)	Overall	p
Total SFR (%)	71.4	84.1	83.3	0.013*
SFR by size (%)· 0–10 mm· 10–20 mm· 20–30 mm	80.075.053.8	96.282.662.0	95.082.061.0	0.198
Operation time ± SD (range), min	60.8 ± 12.5 (32–90)	55.4 ± 14.4 (21–95)	57.5 ± 13.6 (21–95)	p = 0.604
Hospitalization period ± SD (range) days	1.1 ± 1.3 (0–20)	1.4 ± 1.8 (0–12)	1.1 ± 1.3 (0–20)	0.200
Complication total (n)· Clavien 1–2· Clavien 3–4a	4 (7.1%)31	10 (1.5%)73	14 (2.0%)104	0.019**0.689

SFR: Stone-free rate.*Chi-square test. **Fisher’s exact test.

## 4. Discussion

RIRS has become an increasingly popular treatment for renal calculi. The role of RIRS as the primary procedure in treating renal calculi measuring less than 20 mm is becoming more prominent with continuous technical improvements to the size of the scope, degree of deflection, and quality of the fiber optics [11]. Also applied successfully with stones larger than 20 mm. European Association of Urology Guidelines recommends RIRS as the first treatment option for stones smaller than 20 mm and the second option for larger stones. A study by Al Busaidy et al. presented their experience of RIRS in the management of 20–40 mm renal stones. The authors included 71 patients with 20–40 mm renal stones in their study. The authors reported that, the mean number of procedures per patient was 2.1 and the overall SFR was 81%. The authors concluded that the study further supports RIRS as a safe and effective treatment option for 20–40 mm renal stones. Additionally the authors emphasized that, although both the European Association of Urology (EAU) and American Urological Association do not currently recommend RIRS as the first-line treatment of such stones, it appears to be emerging as a commonly utilized primary modality [12]. Therefore, in our clinic, we usually prefer RIRS for non-lower calyceal stones up to 30 mm. Having a lower complication rate than PNL and higher stone-free rate than SWL, RIRS has become one of the most widely adopted methods. In the literature, the stone-free rates after RIRS are reported to range from 47% to 93.3% [11–15]. This wide range of values may be due to the lack of a standard definition for the radiological imaging method, surgeon experience, availability of surgical equipment and the size of the residual stones post-operatively. In 2014, the Clinical Research Office of the Endourological Society published the results of RIRS treatment performed in patients with single kidney stones. A total of 1210 patients were included in that multi-centered study and were divided into three groups according to stone sizes being smaller than 10 mm, 10–20 mm, and greater than 20 mm. Postoperative stones larger than 1 mm were accepted as residual stones and the stone-free rate was calculated as 90.5%, 76.9%, and 31.4% for the three groups, respectively with statistically significant differences. In patients with stones larger than 20 mm, the need for a postoperative JJ stent, postoperative fever, unplanned visit to the hospital after discharge, and hospitalization rates were found to be statistically higher compared to the remaining patients with smaller stones [16]. In our study, the largest stone size was 3 cm. Although it is known that the success rates have decreased, in some cases we preferred this method in patients who were not suitable for open surgery or percutaneous surgery.

In the current study, we evaluated the stone-free status of our patients at four-six weeks after surgery using low-dose contrast enhanced spiral CT. The stone-free rate of all patients obtained in a single session was 83.3%. When the results were evaluated according to stone size, Group 2 with no history of open or percutaneous renal stone surgery was found to have lower stone-free rates as the stone size increased. Nevertheless, group 1 patients had lower stone free rate in all diameter of stone size when compared to Group 2. In other words, in Group 1, the rate of residual stone detection was higher independent of stone size.

Various complications may encountered during and after RIRS. This surgical procedure is not as innocent as expected. Complications associated with infectious processes or ureteral damage are the most frequently reported [17]. As with other endourological procedures, urinary infections should be treated with appropriate antibiotics and the operation should be performed when the urine is sterile [18]. The most frequently encountered complication of this procedure is postoperative infection and despite prophylactic antibiotic treatment. Although no definitive safe operating time has been determined, as the operation time increases, postoperative infective events risk increases. A study by Demir et al., the authors reported that prolonged operation time was an independent risk factor for postoperative fever/infection and the authors found that infective complications increased 11.1-fold during the operation period exceeding 61 min [19]. In our study, the mean operation time was 60.8 ± 12.5 min in a patients underwent open or percutaneous renal stone surgery. According to our results in two patients with septicemia in this group, the operation duration time was 65 and 90 min and this finding was consistent with study of Demir et al. One of the rare complications in RIRS is subcapsular hematoma. In a study conducted by Campobasso et al. [20] it was emphasized that subcapsular hematoma is a rare but important complication. Since we do not perform routine immediate postoperative imaging, we do not know our exact subcapsular hematoma rate. However, Grade 4 renal laceration and subcapsular hematoma associated with about 5 cm retroperitoneal hematoma was detected in and completely resolved by conservative treatment in one of our cases who had open or percutaneous renal stone surgery history. In the current study, postoperative complications were only observed in 14 of our cases (2%). The total complication rate was statistically significantly higher in the group with a history of open or percutaneous surgery; however, there were no significant differences within the two group in terms of subtypes of complications. Grade 1–2 complications were seen in 10 cases and grade 3–4a complications in four patients. Fever was detected in two patients in Group 1 and five patients in Group 2, and was treated using appropriate antibiotics. Septicemia developed in one patient in Group 1 and three patients in Group 2, with one patient from each group requiring intensive care and hospitalization that lasted up to 20 days. RIRS may also have a lower complication rate than PNL, but it should be kept in mind that rare fatal events may occur. Cindolo et al. reported six fatal cases by six urologists. In their study, four patients died from urosepsis, one due to an anesthetic, and one due to hemorrhagic complication. The authors concluded that, despite the fact that RIRS has become a viable option for the treatment of the majority of kidney stones, its complication rates remain low. Nevertheless, rare fatal events may occur, especially in complex cases with a history of urinary tract infections, and advanced neurological diseases [21]. In the current study, we did not have a fatal complication in our patients.

Today, only 1%–5.4% of patients with urinary tract stones require open surgery [22]. According to the EAU guidelines, open surgery is indicated in cases where treatment with invasive methods fail; e.g., staghorn calculi, large stone of mass, complex collecting systems, morbid obesity, and skeletal and/or kidney abnormalities anomalies, as well as in patients with impaired renal function such as nephrectomy, partial nephrectomy European Association of Urology Guidelines on Urolithiasis (2018). pp. 30-31. [online]. Website_https://uroweb.org/wp-content/uploads/EAU-Guidelines-on-Urolithiasis-2018-large-text.pdf [accessed 22/12/2019].1. Although Margel et al. [23] reported an increase in the number of percutaneous interventions following the disruption of intrarenal anatomy due to a history of open surgery (2.3 vs. 1.2), other researchers did not find a significant difference in the average number of interventions between the patients with and without a history of open stone surgery [24]. In the current study, we found lower stone-free rates in patients with a history of open or percutaneous surgical intervention. We attributed this to the impaired renal anatomy making it difficult to access the stone and reducing the possibility of spontaneous stone passage after surgery. Although the stone-free rate decreased as the stone size increased in patients without a history of surgery, a similar difference was not observed in those that had previously undergone open or percutaneous stone removal surgery. This supports our impression that impaired renal anatomy affects stone-free rates regardless of the size of the stone. In the present study, 56 of 707 patients had a history of open or percutaneous renal stone surgery. The stone free rates lower these patients group than the other group (71.4% vs. 84.1%, p = 0.013) and total complications rates are higher than the patients who had no open or percutaeous renal surgery history group (7.1% vs. 1.5%, p = 0.019). 

Alkan et al. evaluated that retrograde intrarenal surgery outcomes in patients who previously underwent open renal stone surgery. The authors compared 32 patients who had undergone open kidney stone surgery and 38 patients who did not. After the first procedure, the stone free rate was 80% and 90%, respectively. There were 17% minor complications in both groups, and they found no difference between the groups in terms of complication rate. The authors reported that they found no major perioperative complications. In conclusion, the authors concluded that RIRS can be safely and effectively performed with acceptable complication rates in patients treated previously with open renal stone surgery [25]. In another study, Osman et al. reviewed 53 patients who underwent RIRS for renal calculi following prior open surgery for urolithiasis. The overall stone-free rates after one and two-procedures were 79.2% and 92.4%. Major complications reported in two patients (3.8%). The authors stated that ureteroscopic retrograde intrarenal surgery for renal calculi following prior open renal surgery was a minimally invasive, safe procedure with a high success rate [26]. Another study by Baylan et al. [27], the authors assessed the efficiency and reliability of retrograde intrarenal surgery secondary to open surgery for kidney stone treatment. Total 120 patients were included in their study. Those who had underwent open surgery, PNL, RIRS, and primary treatment were divided into Group 1, Group 2, Group 3, and Group 4 respectively. The authors found no statistically significant difference in terms of success, hospitalization and complications among the groups. The authors concluded that RIRS is an efficient and safe method for kidney stone treatment of the patients with previous history of open surgery, percutaneous nephrolithotomy and retrograde intrarenal surgery. Similar to these studies, in the current study we found that RIRS effective and safe method for kidney stone treatment with previous history of open surgery and percutaneous nephrolithotomy. But unlike to the Alkan and Baylan’s study, we found that the stone free rate in these patients was lower than the other group. We claim that our unfavorable outcomes related to open or percutaneous renal stone surgery can have an adverse effect on the collecting system of the kidney.

There are a few limitations of our study. The first one, study design is the retrospective and a difference between the groups in terms of number of patients. The second one is the lack of more detailed imaging in patients with a history of open and percutaneous renal surgery. Finally, the lack of data on whether there are problems about deflection or flexion of flexible ureterorenoscope device during the operation.

In conclusion, open or percutaneous renal stone surgery may lead to distortion of the kidney collecting system. According to our results, having a history of open or percutaneous renal stone surgery negatively affects the success and complication rates in RIRS. Therefore, patients should be well informed before the operation.

## References

[ref1] (2013). gender‐specific aspects in urolithiasis. World Journal of Urology.

[ref2] (2014). Changing trends in the American diet and the rising prevalence of kidney stones. Urology.

[ref3] (2011). Tepeler A et al. Urological Research.

[ref4] (2005). Life style and nutritional habits in cases with urinary stone disease. Turkish Journal of Urology.

[ref5] (2007). Extracorporeal shock wave lithotripsy (ESWL): a chronology. Journal of Endourology.

[ref6] (2013). Comparison of retrograde intrarenal surgery, shockwave lithotripsy, and percutaneous nephrolithotomy for treatment of medium-sized radiolucent renal stones. World Journal of Urology.

[ref7] (2010). Retrograde intrarenal surgery for renal stones smaller than 2 cm. Singapore Medical Journal.

[ref8] (2012). Comparison of percutaneous nephrolithotomy and retrograde flexible nephrolithotripsy for the management of 2–4 cm stones: a matched-pair analysis. BJU International.

[ref9] (2019). Changing trends in the treatment of nephrolithiasis in the real world. Journal Of Endourology.

[ref10] (2004). Classification of surgical complications: a new proposal with evaluation in a cohort of 6336 patients and results of a survey. Annals of Surgery.

[ref11] (2009). Retrograde ureteroscopy for renal stones larger than 2.5 cm. Journal of Endourology.

[ref12] (2016). Is RIRS emerging as the preferred option for the management of 2 cm-4 cm renal stones: our experience. Canadian Journal of Urology.

[ref13] (2004). Combined electrohydraulic and holmium: YAG laser ureteroscopic nephrolithotripsy for 20 to 40 mm renal calculi. Journal of Urology.

[ref14] (2008). Flexible ureteroscopy and laser lithotripsy for single intrarenal stones 2 cm or greaterdis this the new frontier?. Journal of Urology.

[ref15] (2009). Percutaneous nephrostolithotomy versus flexible ureteroscopy/ holmium laser lithotripsy: cost and outcome analysis. Journal of Urology.

[ref16] (2015). Outcomes of flexible ureterorenoscopy for solitary renal stones in the CROES URS global study. Journal of Urology.

[ref17] (2018). Flexible ureteroscopy for kidney stones in the third millennium: lights and shadows. Minerva Urologica E Nefrologica.

[ref18] (2001). Ureteroscopic management of lower-pole renal calculi: technique of calculus displacement. Journal of Endourology.

[ref19] (2019). Ayyildiz A et al. Journal of The College of Physicians and Surgeons Pakistan.

[ref20] (2018). Subcapsular renal hematoma after retrograde ureterorenoscopic lithotripsy: our experience. Minerva Urologica E Nefrologica.

[ref21] (2016). Mortality and flexible ureteroscopy: analysis of six cases. World Journal of Urology.

[ref22] (2006). Is there still a role for open surgery in the management of renal stones. Current Opinion In Urology.

[ref23] (2005). Percutaneous nephrolithotomy in patients who previously underwent open nephrolithotomy. Journal of Endourology.

[ref24] (2007). Does previous open nephrolithotomy affect the outcome of percutaneous nephrolithotomy?. Journal of Endourology.

[ref25] (2015). Retrograde intrarenal surgery in patients who previously underwent open renal stone surgery. Minimally Invasive Surgery.

[ref26] (2012). Ureteroscopic retrograde intrarenal surgery after previous open renal stone surgery: initial experience. Urological Research.

[ref27] (2020). Is RIRS safe and efficient in patients with kidney stones who had previous open, endoscopic, percutaneous kidney stone surgery? One center retrospective study. Urology Journal.

